# Novel permittivity test for determination of yeast surface charge and flocculation abilities

**DOI:** 10.1007/s10295-012-1193-y

**Published:** 2012-09-14

**Authors:** Dorota Kregiel, Joanna Berlowska, Bronisław Szubzda

**Affiliations:** 1 Institute of Fermentation Technology and Microbiology, Technical University of Lodz, Wolczanska 171/173, 90-924 Lodz, Poland; 2 Division of Electrotechnology and Materials Science, Electrotechnical Institute, M. Skłodowskiej-Curie 55/61, 50-369 Wrocław, Poland

**Keywords:** Yeasts, Flocculation, Surface charge, Permittivity, Biocontrol

## Abstract

Yeast flocculation has been found to be important in many biotechnological processes. It has been suggested that flocculation is promoted by decreasing electrostatic repulsion between cells. In this study, we used an unconventional rapid technique—permittivity test—for determination of the flocculation properties and surface charge values of three industrial yeast strains with well-known flocculation characteristics: *Saccharomyces cerevisiae* NCYC 1017 (brewery, ale), *S. pastorianus* NCYC 680 (brewery, lager), and *Debaryomyces occidentalis* LOCK 0251 (unconventional amylolytic yeast). The measurements of permittivity were compared with the results from two classical methods for determination of surface charge: Alcian blue retention and Sephadex DEAE attachment. The permittivity values for particular strains correlated directly with the results of Alcian blue retention (*r* = 0.9). The results also confirmed a strong negative relationship between the capacitance of yeast suspensions and their flocculation abilities. The highest permittivity was noted for the ale strain NCYC 1017, with weak flocculation abilities, and the lowest for the flocculating lager yeast NCYC 680. This paper is the first to describe the possibility of using a rapid permittivity test to evaluate the surface charge of yeast cells and their flocculation abilities. This method is of practical value in various biotechnological industries where flocculation is applied as a major method of cell separation.

## Introduction

For many industrial applications in which *Saccharomyces* sp. is used, e.g., beer, wine, and ethanol production, appropriate flocculation behavior is certainly one of the most important characteristics of a good production strain. Yeast cell flocculation has been the subject of numerous studies, but knowledge concerning this process is still incomplete. This phenomenon is a very complex process that depends on both the expression of specific flocculation genes such as *FLO1*, *FLO5*, *FLO8*, and *FLO11* and factors that affect cell wall composition [30, 38, 39].

Yeast cell wall makes up between 10 and 25 % of cell volume, being composed mostly of fibrous β-1,3 glucan and mannoproteins, which are extensively O- and N-glycosylated [17, 18]. Phosphorylation of the mannosyl side chains gives yeast its anionic surface charge [6, 20]. Therefore, forces that influence cell-to-cell binding may also include electrostatic interactions [5, 33, 36, 37].

Flocculation is not only stimulated by the makeup of the yeast cell wall, but is also the result of the physical and chemical parameters of the fermentation medium. The degree of flocculation in brewery yeasts depends on the gravity of the wort, temperature, yeast pitching rate, and oxygen content [3]; For example, low temperatures generally promote cell–cell binding, but osmotic and ethanol stress, as well as continuous mild heat shock, may have a negative impact on the phenotypic expression of flocculation [7].

Yeast flocculation has been found to be important not only in brewing but also in other areas, such as medicine (cytodiagnosis, interactions of pathogens with animal host tissues, determination of organic implant acceptance), industry (biofilm formation, contamination), and biotechnology (sedimentation, attachment of yeasts to solid carriers, wastewater treatment) [14, 24, 31, 36].

Several studies have indicated that the cell surface charge changes when flocculation commences; i.e., a decrease in the cell surface charge occurs at the onset of flocculation. It was suggested that such a decrease in cell surface charge promotes flocculation by decreasing the electrostatic repulsion between cells [39]. Microbial surface charge is often determined using electrostatic chromatography by measurement of the electrophoretic motility or determination of the zeta potential [25, 40]. Alcian blue retention (ABR) or Sephadex attachment assays represent other classical methods for determining this parameter [11, 29].

**Fig. 1:**
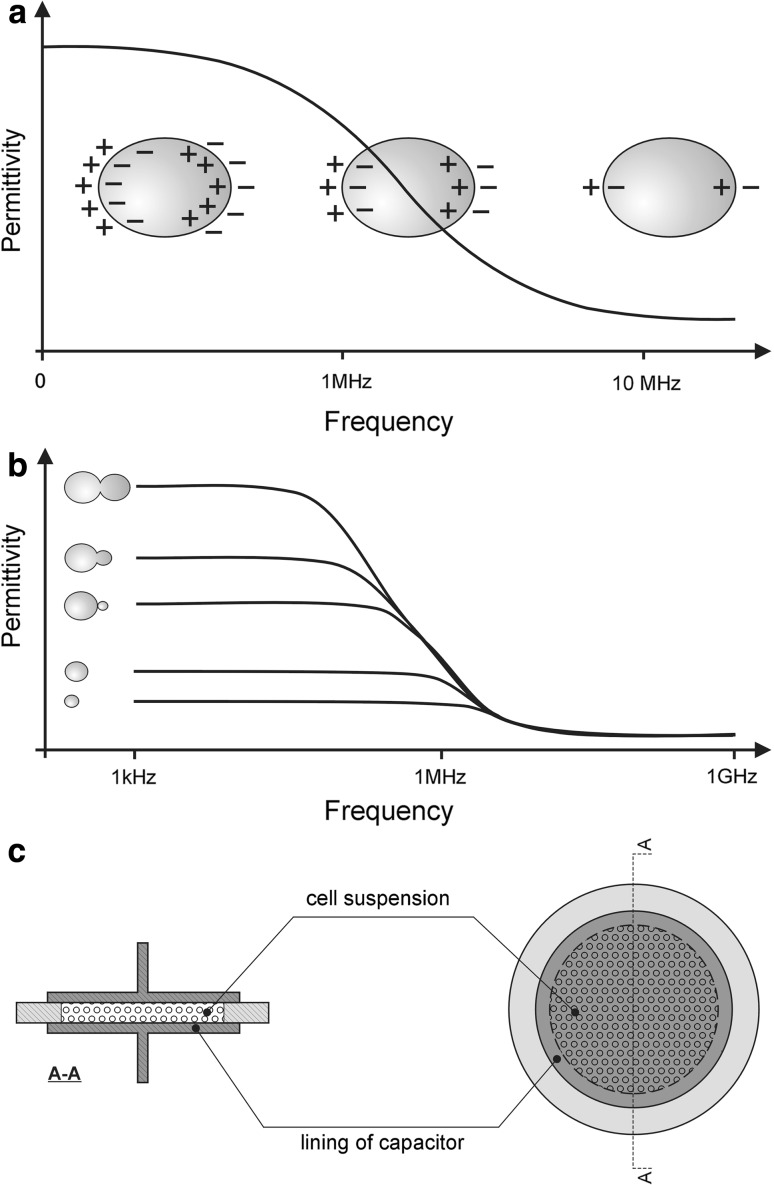
Permittivity of living cell suspensions. **a** Dependence on frequency. **b** Dependence on cell properties. **c** Scheme of flat capacitor used in the study

Yeast cells, due to their surface charge, act as dielectric materials [8, 12, 16, 25]. Numerous studies have demonstrated electrical detection and characterization of the cell surface charge by studying cell attachment to different carbon electrodes or by using combined hydrodynamic flow systems with special impedance spectroscopy techniques [1, 2, 10, 22, 26, 28, 40]. The measurement of the dielectric properties of microbial cell suspensions is based on the ability of biological cells to accumulate charges when exposed to an electrical field. The well-known term “conductivity” reflects the concentration of aqueous ions, their mobility and valence, whilst “permittivity” provides knowledge about the polarization-relaxation response of cells to an external electric field as a function of excitation frequency [9]. The permittivity of living cell suspensions depends on the electrical field frequency, and falls in a series of steps, also called dispersions, as frequency increases [15]. At radiofrequencies, between 0.1 and 20 MHz, the dispersion results from the buildup of charges at cell membranes. A way to interpret this phenomenon is to compare the frequency of the electric field with the rate of cell polarization. At low frequencies (below 0.1 MHz), the field changes direction slowly enough to enable complete polarization of the cells. Accordingly, the measured permittivity is maximal. At high frequencies (above 20 MHz), the cells no longer have time to polarize. The residual permittivity is minimal, and corresponds essentially to the permittivity of the culture medium alone (Fig. 1a) [33]. Permittivity is also closely related to the age, shape, size, chemical composition, and cell density [28, 33, 35] (Fig. 1b). Therefore, valuable insight into the physiology of different eukaryotic cells can be obtained by studying their dielectric properties [2, 4, 9, 13, 15, 22, 23, 33, 43]. The results of these studies stimulated our research.

In this work, permittivity tests were conducted on two brewery yeast strains with different, well-known flocculation characteristics. Additionally, the unconventional strain *Debaryomyces occidentalis* was used as control material. The yeast surface charge was assessed based on an alternative rapid technique that measures the permittivity of yeast cell suspensions. The flocculation properties and surface charge values of the tested strains were compared with the results obtained from two classical methods: ABR and Sephadex attachment.

## Materials and methods

### Yeast strains, media, and culture conditions

In research work, three different strains from the NCYC collection (UK) and the LOCK105 collection (Poland) were used: *Saccharomyces cerevisiae* NCYC 1017 (brewery, ale strain), *Saccharomyces pastorianus* NCYC 680 (brewery, lager strain), and *D. occidentalis* LOCK 0251 (unconventional yeast). The yeasts were stored on wort agar slants at room temperature. Directly, before the experiment, they were activated by passage on fresh agar slants and incubated at 30 °C for 48 h. Propagation of yeasts was done in wort broth (Merck), in 500-ml round-bottomed flask filled with 50 ml medium (pH 5.0) on a laboratory shaker (220 rpm) at temperature of 30 °C for 48 h. After growth, cells were harvested by centrifugation (2,000×*g*) and finally resuspended in deionized water. The number of yeast cells in prepared suspensions was checked by analysis of microscopic images using an Olympus BX41 microscope with digital camera, Thoma counting chamber, and WinMeasure software (version 1.00).

### Evaluation of cell surface charge

Cell surface charge was determined using the ABR assay. Standardized 1 ml yeast suspensions (5 × 10^7^ cells/ml) in 0.02 M sodium acetate buffer (pH 4.0) in silicone tubes (2 ml) were resuspended in 1.8 ml Alcian blue dye (Sigma-Aldrich) buffer solution (50 mg/l; 0.02 M sodium acetate buffer; pH 4.0). The suspensions were incubated for 30 min at 25 °C and centrifuged (25 °C, 10 min, 2,000×*g*), and the amount of free dye remaining in the supernatant was measured spectrophotometrically at wavelength of 615 nm using a SPEKOL 220 spectrophotometer (Carl Zeiss Jena) and compared with a dye standard curve. The surface charge of cells was expressed as ABR equal to the amount of Alcian blue adsorbed by 5 × 10^7^ cells [11].

Additionally, cell surface charge was assessed by attachment to Sephadex DEAE (positive) anion exchanger (Sigma-Aldrich). Samples of 4 ml yeast suspensions (5 × 10^7^ cells/ml) in 0.2 M sodium phosphate buffer were mixed in test silicone tubes with 1 ml Sephadex gel. Cell–bead suspensions were incubated for 30 min at 25 °C with frequent agitation. After shaking, beads and attaching cells were left for 1 min to sediment. Supernatant with nonadherent cells was enumerated with Thoma counting chamber. The cell surface charge was expressed as the amount of cells (%) adsorbed on Sephadex DEAE beads [29].

### Measurement of yeast permittivity

Permittivity tests were carried out in a flat capacitor (Fig. 1c). The measurement chamber had the form of a cylinder 1.8 mm high and 21.2 mm in diameter. All measurements were done at room temperature of 21 °C. The measurements were done using yeast cell suspensions (10^9^ cells/ml) in redistilled water. The control sample was an identical volume of redistilled water, placed in the same testing chamber. The relative permittivity *ε* of the tested yeasts was the ratio of the capacitance *C*_*x*_ of a capacitor in which the space between and around the electrodes is entirely and exclusively filled with the material in question, to the capacitance *C*_0_ of the same configuration of electrodes where the space was filled with the solution without yeasts. The tests were carried out using the QuadTech 1693 RLC Digibridge, microprocessor-controlled, automatic, programmable RLC measuring instrument. The basic accuracy of capacitance measurement was 0.02 %. For all samples, the measurements of condenser capacitance were carried out at a frequency of measurement current of 1 kHz and at a frequency in the range from 100 Hz to 100 kHz. The maximum value of the measurement current voltage amounted to 1 V in all cases. For each frequency a measurement of the comparative sample capacitance was performed [32].

### Statistical analysis

Results are reported as the mean of three independent experiments. Correlation coefficients (*r* values) between the surface charge results obtained using the three different analytical methods were calculated using Microsoft Office Excel 2007.

## Results

**Fig. 2:**
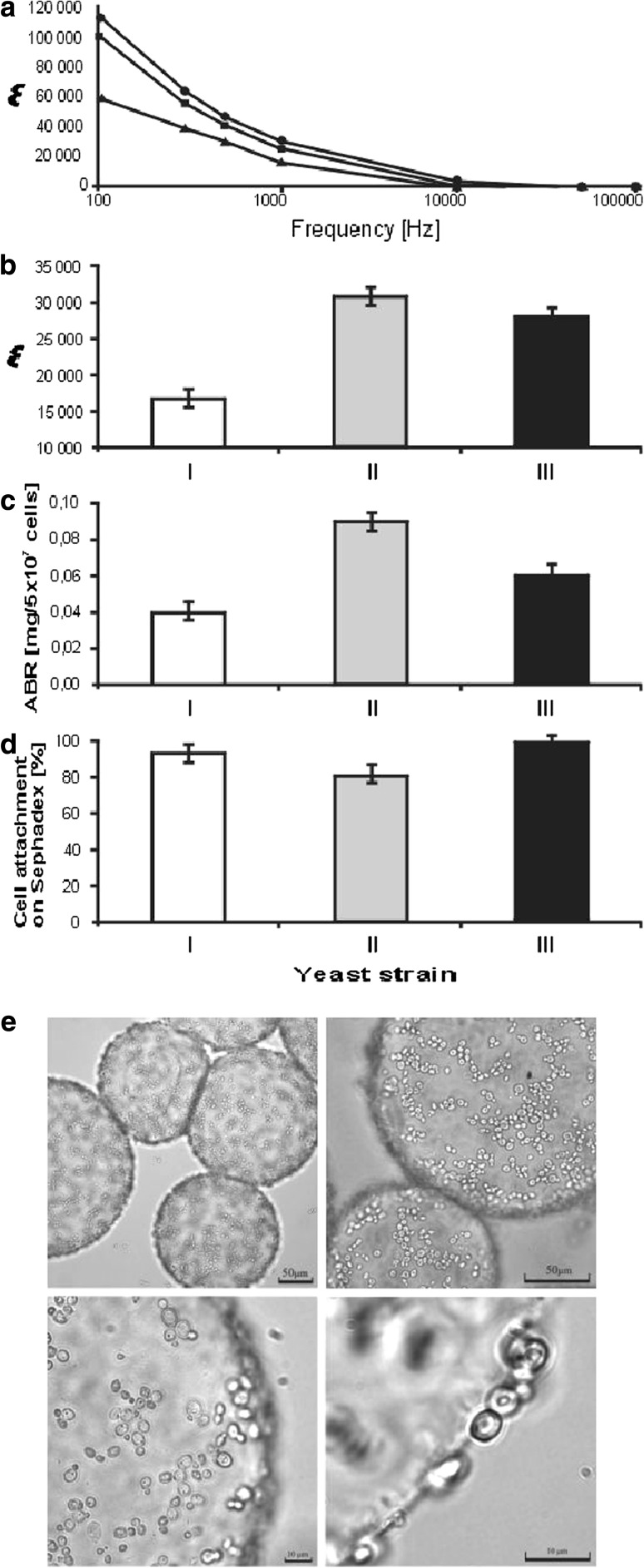
Determination of surface charge of different yeast strains. **a** Dependence of yeast strain permittivity on frequency: *open triangles* NCYC 680, *filled circles* NCYC 1017, *filled squares* LOCK 0251. **b** Permittivity of yeast strains at 1 kHz. **c** Alcian blue retention by yeast cells. **d**, **e** Attachment of yeast cells on Sephadex DEAE beads. *I* NCYC 680, *II* NCYC 1017, *III* LOCK 0251

The series of tests was carried out for all three yeast strains. The permittivity values *ε* were calculated based on the ratio of the capacitance of the capacitor with the tested sample, containing in each case 1 × 10^9^ cells/ml of yeast suspension, to the capacitance of the analogous capacitor without the yeast content. The electrical permittivity of the yeast suspension depended strongly on frequency, reaching the level of 20 for the flocculating lager NCBY 680 strain and 83 for the ale NCBY 1017 strain, at 100 kHz (Fig. 2a). The highest permittivity was noted for the nonflocculating ale strain NCBY 1017, which at 1 kHz reached the value of 3.08 × 10^4^ (Fig. 2b). The *ε* values measured for individual strains showed a strong correlation with the values of ABR (*r* = 0.90). *Saccharomyces cerevisiae* ale strain NCYC 1017 exhibited the highest surface charge (0.09 mg Alcian blue adsorbed per 5 × 10^7^ cells). *S. pastorianus* NCYC 680 strain, described in the catalog of NCBY as a lager yeast, showed the lowest cell surface charge (0.04 mg per 5 × 10^7^ cells). Strain *D. occidentalis* LOCK 0251 was characterized by a medium negative charge (0.06 mg per 5 × 10^7^ cells) (Fig. 2c). The results obtained for these yeast strains with the use of the Sephadex method were not so diverse as in the case of the Alcian blue assay (Fig. 2d). We could observe spatially hindered access of yeast cells to the Sephadex surface (Fig. 2e), which could explain the weak positive correlation found between the yeast permittivity and cell attachment to Sephadex DEAE beads (*r* = 0.37).

## Discussion

Membrane potential is an effect of accumulation of mobile electric charge carriers at membrane surfaces. When living cells are placed in time-oscillating electric fields, these charges move on the membrane surface, giving rise to extremely high polarizations. Within this range, the permittivity of live cell suspensions can be as high as 10^6^ [26, 27]. At frequency <1 GHz or so, the electrical properties of ionic solutions are frequency independent, and may be assessed by measuring the capacitance and conductance of a sample held between two electrodes. In contrast to those of simple ionic solutions, the electrical properties of biological cells generally, and microbial suspensions in particular, are strongly frequency dependent. Additionally, it has been shown that the permittivity is linear with yeast biomass concentration [13]. A linear relationship was also found for the number of viable cells [22]. In this study, we confirmed this effect, as the most well-differentiated permittivity results were obtained at very low frequency of 1 kHz for all the tested strains.

Flocculation, a property of the yeast cell wall, is strongly correlated to the physical surface properties of the cell. It is usually observed at the end of fermentation. The cell surface charge was previously described as an important factor that promotes yeast flocculation. A decrease in the cell surface charge was suggested as a factor promoting flocculation by decreasing the electrostatic repulsion between cells [30, 38]. This was confirmed in our study for different selected brewery yeast strains with specific flocculation characteristics. In the experiments, the concentration of yeast cells, kind of medium, temperature, and phase of growth were kept the same. Therefore, we can suppose that the membrane potential of the tested yeast strains was varied and could influence the results of the permittivity test.

Adsorption of positively charged Alcian blue to yeast cells is a typical electrostatic interaction. Thus, the ABR parameter is an indicator of the overall negative charge of the yeast cell surface [41]. Interestingly, this strong correlation was not observed for the results obtained using another classical test—Sephadex DEAE assay. This may be due to (1) changes in the localization of elementary surface charges as a result of the contact of a cell with Sephadex anion exchanger, and (2) spatially hindered access of yeast cells to the Sephadex surface. This latter supposition was confirmed by the microscopic images.

Different dielectric methods provided fast immediate information about cell concentration, changes in cell volume, and cell viability [1, 8, 21, 25]. In recent years, permittivity has been exploited for the development of novel bioinstrumentation. This measurement has been widely used in medicine to differentiate normal from malignant tissues and to determine the state of different organs [10]. In biotechnology, one of the major applications was in online measurement of cellular biomass during fermentation [9, 19] or in control of cell death in stress conditions [33]. Additionally, permittivity can be used as a highly sensitive separation method for isolation of particular cell types [34].

Since the cell concentration increases exponentially with cell growth and levels off at the stationary phase, the relative permittivity of the yeast culture in broth showed an exponential increase followed by a plateau. Therefore, the trace of permittivity was similar to typical growth curves. In whisky fermentation, the changes in relative permittivity of the fermenting wort showed four distinct phases. In the first phase, the permittivity increased owing to the increase in the cell number. After the increase in the cell number stopped, an increase in *ε* was still observed (the second phase), being explained in terms of the increase in cell volume. In the third phase, there was a decrease in *ε* due to both the decrease in cell volume and the increase in the number of lifeless cells. In the final phase the relative permittivity became the same value as that of the medium, indicating that most cells were defunct because dead cells with leaky plasma membranes are not polarized. In beer fermentation, dielectric monitoring suggested that cells were alive throughout fermentation and that cell growth was highly synchronized [1].

The relationship between dielectric properties and viable cell count was examined, demonstrating that the definition of viability was critical when analyzing biomass online. The results obtained by Opel et al. [22] indicated that the assumptions of dielectric properties were not valid during cell processes. Different dielectric characteristics of intra- and extracellular medium (e.g., ion concentration, presence of organelles) or cell size and shape still have a measurable influence on the dielectric spectrum [15]. Among other possible mechanisms leading to variations of the internal conductivity, the role of trehalose and glycogen deserves some attention: these sugars are accumulated in fairly large amounts by *S. cerevisiae*, either as reserve carbohydrates before entering the stationary phase, or as heat-protecting agents. As storage materials they can both represent up to 30 % of cell dry weight, equivalent to an average intracellular concentration of 150–300 g/l, able to affect the cytosol viscosity and ion mobility. Finally, the intracellular pH could also play a role, since it modulates the level of protonation and hence the charge of molecules with ionizable functions. It is well accepted that the intracellular pH varies with the culture phase: being close to neutrality in exponential phase, it tends to balance the pH of the medium during lag phase or stationary phase [33]. Additionally, the major source of the nonlinear dielectricity may be also H(+)-ATPase [42]. The activity of this enzyme depends on different cell-associated and environmental factors. However, the findings demonstrated that dielectric methods, which are not a substitute for viable cell counts, may be a complementary measurement of workable biomass, providing useful auxiliary information about the physiological state of a culture.

## Conclusions

The obtained investigation results confirm the initial presumptions made by the authors that there is a dependence between the permittivity of brewery strains and their flocculation abilities. These preliminary studies can be a source of inspiration for future studies on the application of permittivity tests for assessing the flocculation ability of different yeasts. The authors will monitor the permittivity characteristics of industrial yeast strains under conditions similar to those used in fermentation processes to confirm the results obtained in model conditions using appropriate time-oscillating electric fields. The first results may provide a base to consider that this unconventional method of surface charge determination can be used not only in brewery industry, but also in production of other alcoholic beverages, as well as in production of biofuels, in modern biotechnology, and in numerous other applications where flocculation is used as an important process of cell separation.
